# Towards a Functional Cure of HIV-1: Insight Into the Chromatin Landscape of the Provirus

**DOI:** 10.3389/fmicb.2021.636642

**Published:** 2021-03-18

**Authors:** Julie Janssens, Anne Bruggemans, Frauke Christ, Zeger Debyser

**Affiliations:** Laboratory for Molecular Virology and Gene Therapy, Department of Pharmaceutical and Pharmacological Sciences, KU Leuven, Leuven, Belgium

**Keywords:** HIV-1, latency, integration site selection, chromatin landscape, block-and-lock, LEDGF/p75, HIV-1 reservoir

## Abstract

Despite potent combination antiretroviral therapy, HIV-1 infection persists due to irreversible integration of the virus in long-living cells of the immune system. The main focus of HIV-1 cure strategies has been on HIV-1 eradication, yet without great success so far. Therefore, HIV-1 remission or a functional cure, whereby the virus is silenced rather than eradicated, is considered as an alternative strategy. Elite controllers, individuals who spontaneously control HIV-1, may point us the way toward a functional HIV-1 cure. In order to achieve such a cure, a profound understanding of the mechanisms controlling HIV-1 expression and silencing is needed. In recent years, evidence has grown that the site of integration as well as the chromatin landscape surrounding the integration site affects the transcriptional state of the provirus. Still, at present, the impact of integration site selection on the establishment and maintenance of the HIV-1 reservoirs remains poorly understood. The discovery of LEDGF/p75 as a binding partner of HIV-1 integrase has led to a better understanding of integration site selection. LEDGF/p75 is one of the important determinants of integration site selection and targets integration toward active genes. In this review, we will provide an overview of the most important determinants of integration site selection. Secondly, we will discuss the chromatin landscape at the integration site and its implications on HIV-1 gene expression and silencing. Finally, we will discuss how interventions that affect integration site selection or modifications of the chromatin could yield a functional cure of HIV-1 infection.

## Introduction

Integration of the HIV-1 DNA into the host genome is central in the replication and pathogenesis of HIV-1 infection. Although the majority of cells productively infected with HIV-1 are rapidly eliminated by the host’s immune system, some infected cells contain provirus that enters a silent mode. In the absence of viral protein expression, those cells are not recognized by the immune system and thus persist indefinitely (Finzi et al., 1999). Although combination antiretroviral therapy (cART) reduces plasma viremia below detectable levels, the virus inevitably rebounds after cART is discontinued (Chun et al., 1997). As such, the persistence of the latent proviruses in long-living cellular reservoirs remains one of the major hurdles to cure HIV-1 infection (Deeks et al., 2016).

The high complexity of the latent reservoirs fuels ongoing discussions on the mechanisms that contribute to HIV-1 persistence. Latent HIV-1 provirus resides in many cellular and anatomical reservoirs, adding to the unknowns of HIV-1 persistence (Barton et al., 2016). Long-living memory CD4+ T cells are considered as the primary cell types constituting the latent reservoirs (Barton et al., 2016). In addition to memory CD4+ T cells, monocyte-derived macrophages are potential important long-lived HIV-1 reservoirs (Kruize and Kootstra, 2019; Wong et al., 2019). Studies in animal models have shown that macrophages sustain infection and are a source of viral rebound after cART interruption (Honeycutt et al., 2017; Abreu et al., 2019). Replication-competent proviral DNA has been isolated from urethral macrophages (Ganor et al., 2019). Still, macrophages are widely distributed in many different tissues and organs, making them difficult to isolate from patients. Therefore, it remains unknown to which extent they contribute to long-lived HIV-1 reservoirs in patients on treatment.

Persistent viremia in cART-treated individuals can originate from antigen-mediated reactivation of memory cells, initiating new rounds of infection. Although several reports indicate that the latent reservoir is maintained by ongoing viral replication owing to limited drug penetration in some anatomical sites (Fletcher et al., 2014; Lorenzo-Redondo et al., 2016), the absence of viral sequence evolution in some patients questions this hypothesis (van Zyl et al., 2018). Furthermore, attempts to reduce the size of the reservoir by treatment intensification have failed, minimizing the contribution of ongoing replication to the total HIV-1 reservoir (Lafeuillade, 2012). Other than having a long half-life, memory CD4+ T cells are extended through homeostatic proliferation (Chomont et al., 2009), and they have the capacity to undergo clonal proliferation (Murray et al., 2016; Lee et al., 2017). What is not yet clear is the total contribution of the clonally expanded population to the total reservoir. If clonal expansion is a determining factor maintaining the pool of latently infected cells, understanding its underlying mechanisms will be of paramount importance to identify new approaches that aim to purge the latent reservoir.

The functionality of latent reservoirs is in large part determined by the intactness of the proviral sequences. Therefore, efforts were made to develop more sensitive and accurate assays to obtain full sequencing of the HIV-1 genome in patient samples (Lambrechts et al., 2020). Remarkably, only 10% of the proviral sequences exhibit an intact genome and are thus theoretically able to produce replication-competent virus. Still, the frequency of cells harboring intact viral genomes is notably larger than the actual number of cells that can be stimulated to produce replication-competent viral particles (Bruner et al., 2016). What remains unclear is how to distinguish an intact-replication-competent provirus from an intact, but non-inducible provirus. In the context of a functional HIV-1 cure, this remains an extremely important question to answer.

Eradicating the latent reservoirs is the main focus of the most prominent HIV-1 cure strategies (Deeks et al., 2016; Darcis et al., 2017). The first reported case of a complete HIV-1 eradication was Timothy Ray Brown or the so-called Berlin patient (Hutter et al., 2009), who got infected with HIV-1 and was diagnosed with acute myeloid leukemia. The Berlin patient received a complete immune system replacement through an allogenic Hematopoietic Stem Cell Transplantation (HSCT) from a donor carrying a homozygous deletion in the CCR5 co-receptor gene (CCR5Δ32). This deletion renders CD4+ T cells resistant to most HIV-1 strains. However, HSCT is associated with high mortality rates and therefore not widely applicable to cure all HIV-1 patients. In addition, CCR5Δ32 donors are scarce. Alternatively, autologous hematopoietic stem cells have been engineered with gene-editing technologies (e.g., Zinc-finger or CRISPR-Cas) to delete the CCR5 gene (Tebas et al., 2014). Others aim to directly disrupt the provirus using a CRISPR-Cas approach (Liao et al., 2015). Unfortunately, delivery of gene-editing constructs to all reservoir cells *in vivo* remains a formidable hurdle and gene-editing strategies may suffer from unknown off-target risks (Liao et al., 2015; Wang et al., 2016). Additionally, the “shock and kill” HIV-1 cure strategy aims to eradicate the entire latent reservoir by reversing its latent state (Darcis et al., 2017; Abner and Jordan, 2019). Latency-reversing agents (LRAs) are given to deliberately reactivate proviral transcription in latently infected cells. The hypothesis behind this approach is that reactivated cells will express viral proteins, allowing them to be recognized and destroyed by the host immune system or die due to viral cytopathic effects. On the contrary, long-term HIV-1 remission or a functional cure constitutes an alternative strategy that has recently gained more interest (Darcis et al., 2017; Kessing et al., 2017; Debyser et al., 2019; Moranguinho and Valente, 2020; Vansant et al., 2020a). A functional cure could be achieved by durably silencing the latent provirus in infected cells and thereby preventing viral rebound by creating a cellular reservoir resistant to reactivation.

To achieve such a functional cure, however, it will be necessary to broaden our understanding on the molecular mechanisms contributing to HIV-1 silencing. How the chromatin environment affects proviral gene expression is only partially understood. Many studies have shown that epigenetic silencing can contribute to the establishment of HIV-1 latency (Pearson et al., 2008; Tyagi et al., 2010; Gallastegui et al., 2011; Matsuda et al., 2015; Nguyen et al., 2017). Poorly investigated is the impact of HIV-1 integration in the host chromatin and in particular the impact of integration site selection on HIV-1 gene expression. Although no consensus exists about its relative importance, integration defines the 3D nuclear localization, the chromatin landscape, and the gene in which the provirus ends up. HIV-1 integration is not random, but instead controlled by a complex interplay between viral components and the human genome. Integration is biased toward gene-dense regions and active transcription units (Jordan et al., 2001; Schröder et al., 2002; Maxfield et al., 2005; Barr et al., 2006; Albanese et al., 2008; Dieudonné et al., 2009; Marini et al., 2015; Kok et al., 2016) whereby methylated lysine 36 residues in histone 3 (H3K36me2 and H3K36me3) are typical markers associated with actively transcribing genes (Bannister et al., 2005). The chromatin reader LEDGF/p75 is responsible for interaction of the virus with these epigenetic markers (Eidahl et al., 2013).

In this review, we will first give an overview of the most important factors that determine integration site selection. Next, we will discuss how the chromatin landscape at the integration site affects the establishment of HIV-1 latency. Furthermore, we will discuss how an increased understanding of integration site selection could open new perspectives on the road toward a functional cure for HIV-1 infection.

## Determinants of Integration Site Selection

**FIGURE 1:**
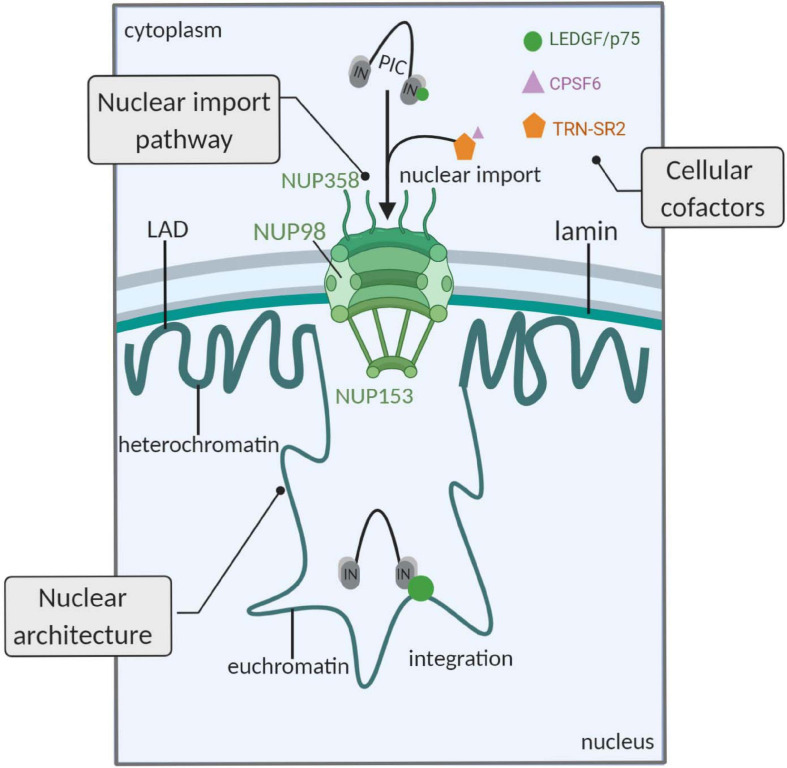
Determinants of HIV-1 integration site selection. The HIV-1 nuclear import pathway is linked to integration. The HIV-1 PIC engages NUPs, favoring integration in close proximity to the NPC (upper left part). Second, cellular cofactors affect the proviral integration site. LEDGF/p75 interacts with HIV-1 IN and targets integration toward active genes. The HIV-1 PIC interacts with other cellular cofactors that affect integration: TRN-SR2 and CPSF6 have been associated with integration in gene-dense regions. TRN-SR2 mediates nuclear import of SR proteins, such as CPSF6, and interacts with the viral IN. The exact mechanism by which these proteins affect nuclear import and integration is not completely understood yet (upper right part). Third, the nuclear architecture affects integration site selection. The heterochromatin condensed regions in the LADs are disfavored, while gene-rich and euchromatin regions in close association with the NPC are frequently targeted by HIV-1 (lower part). PIC, pre-integration complex; NUPs, nucleoporins; LEDGF/p75, lens-epithelium-derived growth factor; IN, integrase; TRN-SR2, transportin-SR2; CPSF6, cleavage and polyadenylation specificity factor 6; LAD, lamina-associated domains; figure created with BioRender.com.

HIV-1 integration is not random. It was shown several years ago that HIV-1 preferentially integrates into active genes (Schröder et al., 2002). The virus has evolved to hijack cellular pathways to target its pre-integration complex (PIC) to active transcription units. Prior to integration of the viral DNA, passage through the nuclear pore is critical to complete the early steps of HIV-1 infection (Figure 1). Correct trafficking through the nuclear pore facilitates subsequent integration into the nuclear periphery in active chromatin (Albanese et al., 2008). Interestingly, genes frequently targeted for HIV-1 integration are closely associated with nuclear pore complexes (NPCs; Demeulemeester et al., 2015). These observations provide evidence that the HIV-1 nuclear import pathway is directly coupled to preferential integration site selection. Furthermore, several NPC-associated proteins influence the nuclear localization of integration. Depletion of Nup98, Nup153, Transportin-SR2 (TRN-SR2, TNPO3), or RanBP2 has been shown to hamper integration in gene-dense regions (Ocwieja et al., 2011; Di Nunzio et al., 2013; Lelek et al., 2015). In conclusion, alternative import pathways but also altered chromatin organization after depletion of certain NPC components may lead to reduced integration gene-dense regions (Lee et al., 2010; Liang and Hetzer, 2011; Di Nunzio et al., 2012; Koh et al., 2013).

Secondly, HIV-1 uses cellular cofactors for retroviral integration site selection (Figure 1; Debyser et al., 2015). HIV-1 integration is catalyzed by the viral integrase (IN) that employs the cellular chromatin reader lens-epithelium-derived growth factor (LEDGF/p75) to target the virus to active transcription units (Cherepanov et al., 2003; Ciuffi et al., 2005; Llano et al., 2006). The C-terminal region of LEDGF/p75 harbors a protein binding domain responsible for interaction with IN (Cherepanov et al., 2005), while the chromatin reading function is executed by the N-terminal PWWP domain (Eidahl et al., 2013). LEDGF/p75 is known to recognize the H3K36me3 mark on nucleosomes (Pradeepa et al., 2012; Eidahl et al., 2013). Upon knockdown of LEDGF/p75, its paralog HRP-2 can substitute for its function as it is the only other identified human protein that possesses both an IN binding domain and a PWWP domain (Schrijvers et al., 2012a, b). Depletion of chromatin tethering cofactors LEDGF/p75 and HRP-2 shifted integration out of active genes, though the integration pattern was not yet completely random, indicating that other host factors are involved (Schrijvers et al., 2012b). Of note, our lab and others have shown that the 3D nuclear localization of HIV-1 integration is affected by LEDGF/p75 (Marini et al., 2015; Vranckx et al., 2016). Following nuclear translocation of the PIC, HIV-1 integrates preferentially in the nuclear periphery in close association to the nuclear pore (Albanese et al., 2008; Dieudonné et al., 2009; Di Primio et al., 2013; Marini et al., 2015). In 2016, it was reported that depletion of LEDGF/p75 or direct interference of small molecule inhibitors (LEDGINs) with the IN-LEDGF/p75 interaction shifts integration toward the inner nucleus compared to wild-type integration (Vranckx et al., 2016).

Additionally, cleavage and polyadenylation specificity factor 6 (CPSF6) has been put forward as another HIV-1 cellular cofactor important for integration site selection. CPSF6 is known to promote HIV-1 nuclear entry through interaction with the viral capsid (CA; Lee et al., 2010; Price et al., 2012; Matreyek et al., 2013; Bhattacharya et al., 2014). Furthermore, depletion and knockout of CPSF6 decreased integration in active genes (Chin et al., 2015; Rasheedi et al., 2016; Sowd et al., 2016; Achuthan et al., 2018). In agreement, introduction of an HIV-1 CA mutation at the CPSF6 binding site (N74D) resulted in more integration in gene-poor regions (Schaller et al., 2011). It was shown that the CA-CPSF6 interaction is necessary to allow integration in transcriptionally active euchromatic regions of the inner nucleus. Against the more prevailing view (Albanese et al., 2008; Dieudonné et al., 2009; Di Primio et al., 2013; Marini et al., 2015), the study of Achuthan et al. (2018) suggested that HIV-1 does not preferentially integrate in the nuclear periphery. Furthermore, they propose that CPSF6, and not LEDGF/p75, is the major regulator of HIV-1 integration site selection. Moreover, the overall contribution of CPSF6 to HIV-1 replication is still under debate since the depletion of endogenous CPSF6 has no major impact on HIV-1 replication in cell lines (Lee et al., 2010; Fricke et al., 2013), in stark contrast to LEDGF/p75 (Vandekerckhove et al., 2006). Nevertheless, it would be of interest to verify whether depletion of CPSF6 also affects immediate latency and reactivation of the provirus, as discussed in the next section.

Adding to the complexity, the dynamic nuclear architecture contributes to the selection of genomic sequences in which HIV-1 integrates (Figure 1). HIV-1 is known to bypass the heterochromatin condensed regions in lamin-associated domains (LADs) and also disfavors centrally located transcriptionally active regions (Lusic and Siliciano, 2016). Earlier work has demonstrated that some genes are more frequently targeted for HIV-1 integration and therefore termed HIV-1 recurrent integration genes (RIGs; Marini et al., 2015). In fact, it was observed that RIGs were prevalently localized at the nuclear periphery (Marini et al., 2015). The nuclear periphery contains open chromatin regions that are associated with NPCs. As mentioned, nuclear trafficking through these complexes affects the integration pattern. Recently, Lucic et al. (2019) reported that certain nuclear sub-compartments were more frequently targeted for HIV-1 integration in activated T lymphocytes. This preferential organization of RIGs was linked to the proximity to super-enhancers (SE). Remarkably, the occupancy of similar 3D compartments by HIV-1 and SE explained the observed phenomenon. Although this strengthens the hypothesis that the 3D nuclear architecture of activated T lymphocytes is a pivotal determinant of the HIV-1 integration, no direct evidence for a mechanism whereby integration is targeted to these sites was given. Furthermore, RIGs only represent a fraction (<30%) of all integration sites and therefore these findings cannot be generalized to all HIV-1 integration sites.

## Ledgins Retarget Integration

In 2010, structure-based drug design targeting the interface between HIV-1 IN and its cellular binding partner LEDGF/p75 resulted in the discovery of 2-(quinolin-3-yl)acetic acid derivatives that impair HIV-1 replication (Christ et al., 2010). Inhibitors of different chemical classes share the identical binding pocket on HIV-1 IN and are therefore called “LEDGINs” (Demeulemeester et al., 2014). In literature, LEDGINs are sometimes termed ALLINIs (allosteric IN inhibitors); however, not all allosteric IN inhibitors target the binding pocket occupied by LEDGF/p75 (Demeulemeester et al., 2014). LEDGINs impede the interaction between HIV-1 and LEDGF/p75 by binding to the IN dimer interface and allosterically inhibit the catalytic activity of IN (the so-called “early effect”) (Christ et al., 2012; Kessl et al., 2012). Afterward, it was demonstrated that not only the early steps, but also later steps in the viral replication cycle (the so-called “late effect”) (Christ et al., 2012; Balakrishnan et al., 2013; Desimmie et al., 2013; Jurado et al., 2013; Le Rouzic et al., 2013) are hampered by LEDGIN treatment. Viral particles generated in the presence of LEDGINs show aberrant morphology because of LEDGIN-induced IN multimerization. Morphological defects typically include a delocalized ribonucleoprotein outside the capsid core, resulting in crippled viruses that are defective for reverse transcription, nuclear import, and integration of the provirus. This novel class of inhibitors is explored by several pharmaceutical companies actively pursuing HIV-1 treatment or cure strategies [reviewed in Demeulemeester et al. (2014)].

**FIGURE 2:**
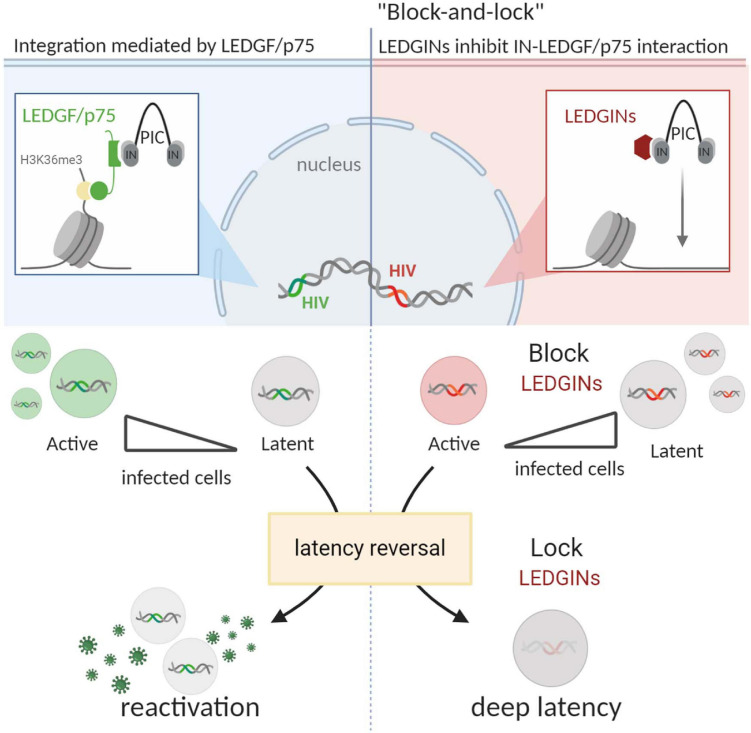
LEDGIN-mediated retargeting provides a “block-and-lock” functional cure. LEDGF/p75 binds to the viral IN and targets the HIV-1 PIC to the chromatin via interaction with H3K36me3 (upper left part). LEDGINs inhibit the LEDGF/p75-IN interaction and retarget integration (upper right part). LEDGF/p75-independent integration in the presence of LEDGINs results in relatively more proviruses that are in a transcriptionally silent or latent state, compared to LEDGF/p75-dependent integration (middle part). After latency reversal, latently infected cells rebound, resulting in reactivation of HIV-1 gene expression (lower left part). In contrast, LEDGIN-mediated retargeting results in a cellular reservoir that is resistant to reactivation after latency reversal. LEDGIN-mediated retargeting provides a potential “block-and-lock” functional cure strategy. LEDGF/p75, lens-epithelium-derived growth factor; IN, integrase; PIC, pre-integration complex; figure created with BioRender.com.

As discussed, LEDGF/p75 is an important determinant of HIV-1 integration site selection, targeting integration toward active regions of the genome (Figure 2). As such, LEDGINs could interfere with the LEDGF/p75-mediated integration pattern. In 2016, such studies were first described by Vranckx et al. (2016). The authors demonstrated that residual HIV-1 integration was clearly shifted out of active genes upon LEDGIN treatment. Furthermore, more provirus was found in the inverse orientation, and the 3D nuclear localization of the provirus was shifted toward the inner nucleus after LEDGIN treatment (Vranckx et al., 2016). Employing a virus with a double reporter designed by the Verdin lab (Battivelli et al., 2018), it was demonstrated that a larger proportion of the provirus exhibited a transcriptionally silent phenotype after LEDGIN-mediated retargeting. Furthermore, this residual reservoir proved less prone to HIV-1 reactivation (Vranckx et al., 2016; Figure 2).

Of note, LEDGINs partially retarget proviral integration in cell culture, even though no random integration is observed. Still, at high concentrations of LEDGINs, nearly all residual replication and reactivation of the latent provirus is blocked. This teaches us that LEDGIN-mediated retargeting surpasses mere integration inside or outside of genes. Instead, by interfering with LEDGF/p75-mediated chromatin recognition of specific epigenetic features, LEDGINs likely affect the epigenetic and chromatin landscape in a more profound manner, resulting in a silent viral reservoir. Further research is required to clarify the mechanism whereby LEDGF/p75 mediates optimal integration site selection. Advancing methods, e.g., branched DNA imaging (bDNA; Puray-Chavez et al., 2017), could further corroborate the impact of LEDGIN-mediated retargeting on the transcriptional state of the provirus at the single-cell level. In conclusion, to ensure both a productive and a latent proviral state, HIV-1 may have evolved to use LEDGF/p75 as a molecular tether. In the absence of LEDGF/p75, the provirus may end up in a deep latent population resistant to reactivation.

## Chromatin Landscape at the HIV-1 Integration Site

To date, it is well established that the HIV-1 promotor is governed by the local chromatin environment (Jordan et al., 2003). The chromatin structure is composed of nucleosomes, consisting of eight core histones that are susceptible to epigenetic modifications. Regardless of the site of integration, two nucleosomes, nuc-0 and nuc-1, are precisely positioned on the viral promotor region downstream of the transcription start site (Verdin, 1991). Active transcription is associated with histone acetylation induced by histone acetyl transferases (HATs). Acetylation loosens the chromatin, rendering the chromatin and in particular promotors more accessible to the transcription factors. In contrast, histone deacetylases (HDACs) are involved in histone deacetylation and usually induce gene silencing. Additionally, DNA methylation at CpG dinucleotides near the viral promoter is associated with HIV-1 latency. CpG methylation has been shown to hamper reactivation in cell lines and patient samples by restricting the accessibility of interacting proteins to the chromatin (Blazkova et al., 2009; Trejbalová et al., 2016). The above findings suggest that HIV-1 gene expression is regulated by the chromatin context surrounding the integration site. Still, at present, studies that have investigated the impact of the chromatin environment on acute or latent HIV-1 infection are scarce.

As mentioned, LEDGINs provide us with an important research tool to study HIV-1 latency. The study by Vranckx et al. (2016) demonstrated that LEDGINs retarget integration toward silent genes that disfavor HIV-1 expression. Still, it was not clear whether the complex nature of the chromatin landscape surrounding the insertion site of those retargeted viruses could explain their silent state. Therefore, B-HIVE technology was employed. By tagging the viral genome with a unique barcode, B-HIVE allows to link individual proviral integration sites with transcriptional levels (Vansant et al., 2020b). As such, expression levels of each single provirus can be correlated to the corresponding integration site. With B-HIVE technology, it was shown that LEDGINs shift the distribution of integration sites out of gene-rich chromosomes, such as chromosomes 16, 17, and 19. In agreement with Vranckx et al. (2016), the residual provirus was retargeted toward silent genes and intergenic regions upon LEDGIN treatment. Furthermore, LEDGINs decreased total RNA expression and enriched the proportion of “no-expression” barcodes, which are silent barcodes without RNA expression. Interestingly, following LEDGIN treatment, these “no-expression” barcodes were located further away from epigenetic markers linked to active transcription (Med1, CBP, H3K4me1, H3K27ac H3K79me2/3, RNAPII, and H3K4me3) (Vansant et al., 2020b). Likewise, the distance of silent provirus to H3K36me3, the epigenetic feature recognized by LEDGF/p75, increased after LEDGINs supporting the direct link between LEDGF/p75-mediated integration and proviral expression. In contrast, the proximity of epigenetic features associated with enhancers (such as H3K27ac, H3K4me1, or Med1) stimulated transcription independent of LEDGIN-mediated retargeting. These data confirm the pivotal role of LEDGF/p75 in integration site selection (Vansant et al., 2020b). However, they also point toward other, LEDGF/p75-independent mechanisms, such as the proximity of integration to genomic enhancers (Chen et al., 2016).

Notably, besides its role in integration site selection, LEDGF/p75 has been proposed to regulate post-integration latency (Gérard et al., 2015; Gao et al., 2020). Recently, Gao et al. published that recruitment of the repressive Pol II-associated factor 1 (PAF-1) by LEDGF/p75 induces Pol II pausing and thereby promotes viral latency. Although somewhat paradoxical, the same study demonstrated distinct effects of LEDGF/p75 during latency formation and latency reactivation. Upon latency reversal, LEDGF/p75 was shown to recruit MLL1, which in turn displaces PAF1 from the viral promotor, and accordingly induces proviral transcription (Gao et al., 2020).

Additionally, polycomb group proteins (PcG) have recently been implicated in epigenetic repression of HIV-1 transcription (Matsuda et al., 2015; Khan et al., 2018). In particular, Polycomb-repressive complex 2 (PRC2) is believed to induce silencing of the HIV-1 promotor by methylation of H3K27, a repressive chromatin mark. Matsuda et al. (2015) published that trimethylation of H3K27 by PRC2 is associated with transcriptional silencing early during the course of infection.

## Elite Control as Model for a Cure for HIV-1

Although a broadly applicable cure of HIV-1 has not been achieved yet, the identification of a unique population of individuals that spontaneously control the infection provides indispensable information on the mechanisms that allow robust virological control in the absence of treatment (Gonzalo-Gil et al., 2017). Such individuals, referred to as HIV-1 controllers, maintain either undetectable (elite controllers, EC) or low levels (viremic controllers) of viremia, stable CD4^+^ cell counts, and accordingly, no signs of clinical progression (Gonzalo-Gil et al., 2017).

The molecular mechanisms responsible for robust virological control in EC remain poorly understood. It has been documented that the proviral reservoirs of latently infected cells are smaller in EC (García et al., 2017), yet genomes isolated from this specific group have shown a similar extent of genetic variation and replication compared to individuals on cART (Blankson et al., 2007). The hypothesis that EC restrict viral replication independent of the viral fitness has been supported by several studies (Blankson et al., 2007; Buckheit et al., 2012; Gonzalo-Gil et al., 2017). In fact, the degree of defective HIV-1 was shown to be comparable between EC and individuals on cART. Spontaneous viral control has been linked to host gene polymorphisms, among which the protective HLA class I and the CCR5 delta 32 (CCR5Δ32) alleles are most consistently recognized (Antoni et al., 2013). Within the EC that exhibit protective HLA class I alleles, viremic control is usually linked with a broad CD8+ T cell response (Pereyra et al., 2008). However, such T cell responses are not always necessary for long-term viral control (Emu et al., 2008). Furthermore, a loss of virological control has more often been experienced in EC with protective HLA class I alleles compared to EC without (Koofhethile et al., 2016). This underscores the need to pinpoint alternative, more durable, and T cell-independent pathways that are responsible for virological control.

Recently, an alternative hypothesis underlying sustainable, long-term HIV-1 remission in EC was proposed by Jiang et al. (2020). They investigated the HIV-1 reservoir in terms of the chromosomal integration site and discovered that individuals naturally controlling HIV-1 (EC) have more proviral DNA in a silent, deep sleep, mode (Chomont, 2020; Jiang et al., 2020). An increased proportion of the integration sites was found in non-coding regions, such as centromeric DNA and dense heterochromatin gene deserts, regions less permissive for active viral transcription (Jiang et al., 2020). Although the absolute number of proviral DNA sequences was lower in EC, they exhibited relatively more genome-intact viral sequences compared to patients on cART. These data strongly suggest that defective HIV-1 cannot explain the clinical status of EC; they rather control HIV-1 through other, still unknown, mechanisms. Interestingly, Jiang and colleagues found no evidence for preferential targeting of repressive chromosomal regions (e.g., centromeric satellite DNA or KRAB-ZNF genes) in EC, which is consistent with the known preferential integration of HIV-1 in active genes and open chromatin (Jordan et al., 2001; Schröder et al., 2002; Maxfield et al., 2005; Barr et al., 2006; Albanese et al., 2008; Dieudonné et al., 2009; Marini et al., 2015; Kok et al., 2016). Therefore, the elite control is probably not the result of an altered integration site preference during reservoir formation in these patients. More likely, proviral sequences integrated in non-silent regions are gradually cleared by the immune system, while a deep latent reservoir is selected over time (Chomont, 2020; Einkauf et al., 2019). This work holds great promises to obtain a functional cure for HIV-1 in which the virus is still present in the latent reservoirs but never reaches high levels of viral replication. EC may provide the translational evidence for the “block-and-lock” induced cure strategy, which aims to permanently lock the virus into a transcriptionally silent or deep latent state, unable to rebound upon cART interruption.

Jiang et al. (2020) also investigated whether epigenetic modifications surrounding the integration site contributed to the long-term transcriptional repression of intact sequences observed in EC. The frequency of CpG methylation at 500- or 1,000-base-pair distance from the integration site was significantly higher for integrants from EC, compared to patients on cART. Genomic regions more sensitive to DNA methyltransferases might be more prone to facilitate deep latency and thus represent preferential hotspots for long-term persistent proviral sequences (Jiang et al., 2020). Furthermore, genome-intact viral sequences of EC were located closer to epigenetic features associated with silent chromatin (H3K9me3) and located further away from the active transcription mark H3K4me1 (Jiang et al., 2020).

In addition, Jiang et al. (2020) frequently observed identical sites derived from clonally expanded cells in EC. They demonstrated that these clonally expanded cells contained intact and replication-competent virus, which is supported by earlier observations (Symons et al., 2018). Remarkably, more integrants originating from a clonal expanded cell population were found in EC compared to cART-treated individuals. So far, the mechanism underlying these large expansions of identical clones remains elusive. Antigen stimulation or homeostatic proliferation are known drivers of clonal expansion (Chomont et al., 2009; Hughes and Coffin, 2016; Kwon and Siliciano, 2017). Clonal expansion of infected cells has also been linked to the proviral integration site (Maldarelli et al., 2014; Wagner et al., 2014; Cohn et al., 2015). Other studies found recurrent HIV-1 integrations in the BACH2, STAT5B, and MKL2 gene of patient samples (Maldarelli et al., 2014; Wagner et al., 2014; Cohn et al., 2015; Cesana et al., 2017). Notably, all three genes code for cellular transcription factors that regulate cell growth and both MKL2 and BACH2 were previously described as oncogenes (Kobayashi et al., 2011; Flucke et al., 2013). These studies support the hypothesis that integration in certain genes promotes clonal expansion of the infected cell and, as such, contributes to HIV-1 persistence. Nevertheless, more investigation is required to prove a causal association between the site of integration and an effect on proliferation or cell survival.

## Insight Into HIV-1 Integration Site Selection Provides a “Block-And-Lock” Functional Cure

Even potent antiretroviral therapy fails to cure HIV-1 infection due to the persistence of long-lived latently infected cellular reservoirs. Viremia inevitably rebounds upon treatment interruption, and therefore, an HIV-1 cure has been extremely difficult to achieve (Chun et al., 1997; Deeks et al., 2016). The well-documented cases of the Berlin and London patients raised hope that curing HIV-1 infection is on the long-term a feasible target (Hutter et al., 2009; Gupta et al., 2020). These patients have obtained complete eradication of all replication-competent HIV-1 and are considered to be cured from HIV-1. Still, such a sterilizing cure is very challenging to achieve and almost impossible to prove with current technologies (Henrich et al., 2014; Abdel-Mohsen et al., 2020). These factors highlight the importance of novel strategies and identification of alternative target routes that can exhaust the latent reservoir and suppress HIV-1 replication in the absence of treatment. The latency-reversing, “shock and kill” strategy has reached considerable attention due to evidence that LRAs may lead to enhanced control of viral load after a treatment interruption (Archin and Margolis, 2014). LRAs enhance HIV-1 transcription *in vivo*, but so far, it has not been conclusively demonstrated that this indeed leads to immune-mediated killing and a sustained reduction in reservoir size (Archin et al., 2012; Søgaard et al., 2015; Darcis et al., 2017). Adding to the complexity, the rebound capacity of latent provirus is determined by the integration site and the nearby chromatin modifications. Hence, a combination of LRAs will be a necessity to reactivate all latent proviruses (Chen et al., 2016; Battivelli et al., 2018). Besides little to no reduction in reservoir size, this strategy is also associated with broad and uncontrolled activation, resulting in undesired off-target immune reactivation (Abner and Jordan, 2019).

Except for the Berlin and London patients, eradication strategies have not resulted into a cure for HIV-1 so far. Therefore, clinicians and scientists had to reevaluate the definition of an HIV-1 cure. Long-term HIV-1 remission or a functional cure whereby the virus is not eradicated from the body but remains suppressed even in the absence of treatment is therefore considered a more feasible outcome. HIV-1 remission is increasingly used in the field to indicate the goal of long-term undetectable viremia in the absence of treatment. HIV-1 remission is therefore considered as a necessary precursor for the development of an HIV-1 cure. We might argue that EC, which fulfill these criteria, have achieved HIV-1 remission or a functional cure. Hence, the mechanisms responsible for elite control are intensively investigated to gain more insight into the feasibility of such an approach. It is widely accepted, however, that the ability to spontaneously control HIV-1 is the result of a complex interplay between virological and immunological factors. As such, no sole mechanism will explain the phenotype of this heterogeneous group of individuals. Nonetheless, the mechanism proposed by Jiang et al. (2020) holds great promises for the HIV-1 cure field. Chromosomal integration into repressive chromatin is proposed as a crucial factor contributing to HIV-1 remission in EC. This insight is not entirely new; a better comprehension of HIV-1 integration site selection could provide us with new strategies for a functional cure of HIV-1 infection (Debyser et al., 2019).

As discussed, epigenetic regulation of the chromatin structure, an integration site-specific feature, is important in the control and maintenance of HIV-1 latency. Therefore, various LRAs are epigenetic regulators: inhibitors of histone methyl transferase (HMTi), histone deacetylase (HDACi), or DNA methyl transferase (DNMTi) have been tested to reactivate proviral transcription in latently infected cells and patients. However, Battivelli et al. (2018) have reported that no LRA so far could reactivate more than 5% of the total reservoir *in vitro* and hence is potent enough to reduce the reservoir size. Alternatively, durably silencing the reservoir through induction of repressive chromatin modifications could obtain a functional HIV-1 cure. In this regard, PcG proteins could provide promising novel targets for the induction of HIV-1 latency (Khan et al., 2018).

Retargeting HIV-1 integration by LEDGINs creates a deep latent cellular reservoir that is resistant to reactivation. As such, LEDGINs present interesting candidates to be used in a “block-and-lock” functional cure strategy. Although promising, as with all strategies, the “block-and-lock” functional cure strategy faces some challenges. While it is not exactly known when the latent reservoirs are formed, at present, it is clear that they are established early during acute infection. It has been shown that early treatment reduces the size of the reservoir during acute infection (Buzon et al., 2014). Hence, addition of a LEDGIN to early cART regimens could modulate the functionality of the latent reservoirs. If LEDGINs will be tested in clinical trials as antivirals for acute infection, monitoring the reservoir size with quantitative viral outgrowth assay (qVOA) and proviral DNA loads could provide supportive evidence for this hypothesis. In the case of HIV-1 pre-exposure prophylaxis (PrEP), LEDGINs may also be beneficial when added since they may ensure that any residual infection under PrEP treatment results in a deeply latent or non-functional provirus. With regard to chronically infected patients, recent studies now suggest that the majority of the reservoir responsible for HIV-1 rebound might only be established in the year before cART is initiated (Brodin et al., 2016; Abrahams et al., 2020), implying that LEDGINs may also have an effect in a first-line treatment of patients diagnosed years after infection. In the absence of any residual replication when the latent reservoirs have already been established, LEDGINs will fail to modulate the functional reservoir in patients on chronic treatment. Yet, if poor drug penetration by cART at sanctuary sites allows any residual replication, these viruses could be retargeted by LEDGINs, assuming that LEDGIN tissue penetration is optimal. Secondly, repeated treatment interruptions followed by reinitiation of cART including LEDGINs could modulate the functional reservoir in chronically infected patients. Whether enough proviruses can be mobilized upon treatment interruption in a clinical setting remains, of course, to be determined.

The question whether we should shock or lock the latent reservoir has been subject to ongoing debate (Darcis et al., 2017). Likewise, the combination of “lock and shock” presents an alternative, yet unexplored strategy. Addition of LEDGINs to early treatment protocols may already reduce the size of the functional reservoir. Afterward, residual replication-competent virus prone to rebound upon treatment interruption (e.g., integrated close to enhancers) could be eradicated with potent LRAs that induce minimal cellular toxicity. To ensure the establishment of a complete and long-term “deep latency”, it may be of interest to target multiple pathways that promote silencing and prevent viral rebound. Besides retargeting integration with LEGDINs, interference with chromatin remodelers and transcription activators such as Tat are interesting approaches to induce long-term suppression of the virus (Li et al., 2019; Vansant et al., 2020a).

In conclusion, several HIV-1 cure strategies have been extensively studied and evaluated in patients. Unfortunately, none of these approaches was successful so far on a large scale in the clinic. All HIV-1 cure approaches face the same obstacle: the persistence of latent reservoirs carrying replication-competent provirus. In this respect, a better understanding of the mechanisms that contribute to silencing of the reservoirs is of paramount importance. Therefore, a persistent effort is required to increase our basic understanding on the virology of HIV-1. As described in this review, increasing our knowledge on the importance of integration site selection in the establishment and maintenance of the reservoir will certainly help us toward a functional cure of HIV-1 infection.

## Author Contributions

JJ made the figures. All authors wrote, read, and agreed to the published version of the manuscript.

## Conflict of Interest

The authors declare that the research was conducted in the absence of any commercial or financial relationships that could be construed as a potential conflict of interest.
